# Phenotype–genotype correlations in patients with *TGFBI*-linked corneal dystrophies in Taiwan

**Published:** 2012-02-07

**Authors:** Yu-Chih Hou, I-Jong Wang, Cheng-Hsiang Hsiao, Wei-Li Chen, Fung-Rong Hu

**Affiliations:** 1 Department of Ophthalmology, National Taiwan University Hospital, College of Medicine, National Taiwan University, Taipei, Taiwan; 2 Department of Pathology, National Taiwan University Hospital, College of Medicine, National Taiwan University, Taipei, Taiwan

## Abstract

**Purpose:**

To determine the phenotype–genotype correlations in patients with corneal dystrophies associated with human transforming growth factor-β-induced (*TGFBI*) mutations at the National Taiwan University Hospital.

**Methods:**

Twenty-five affected patients from 15 families with corneal dystrophies were recruited. They underwent slit-lamp biomicroscopy and visual acuity examinations. Genomic DNA was extracted from their peripheral blood, and the exons amplified from *TGFBI* were sequenced.

**Results:**

Eleven patients from 9 families with granular corneal dystrophy (GCD) presented with a wide spectrum of dot or fleck opacities and shared some similar clinical features. Genetic studies revealed an R124H mutation in 5 families and an R555W mutation in 4 families. A patient with GCD type 2 and an R124H mutation showed a marked increase in opacities in the laser-assisted in situ keratomileusis (LASIK) flap interface. Six patients from 3 families with superficial honeycomb opacities had an R555Q mutation. Of the 4 patients from 3 families with variant lattice line opacities, 3 from 2 families had an R124C mutation, whereas 1 from the third family had an A546D mutation. Spontaneous mutations were detected in 2 families: an R124C mutation in 1 family with lattice corneal dystrophy (LCD) type I and an A546D mutation in the other with atypical LCD.

**Conclusions:**

In most cases, *TGFBI*-linked corneal dystrophies had good phenotype–genotype correlations; however, some phenotypic variation was present. The most common mutations in Taiwan were R124H in GCD type 2 and R555W in GCD type 1. The R555Q mutation in Thiel–Behnke corneal dystrophy is not as rare in Taiwan as it is in other Asian countries. Sequencing of *TGFBI* can aid in the precise classification of these corneal dystrophies.

## Introduction

Corneal dystrophies are a heterogeneous group of inherited, bilaterally progressive corneal opacities without inflammation. These variable opacities often result in recurrent corneal erosion and visual impairment. Most corneal dystrophies show an autosomal dominant inheritance pattern with a high degree of penetrance. Three autosomal dominant corneal dystrophies, including granular dystrophy Groenouw type I (GCD1), lattice type I (LCD1), and Avellino (ACD or GCD2), have been mapped to chromosome 5q31 [1]. GCD1 is characterized by bread crumb- or snowflake-like opacities that stain positive on Masson trichrome staining [2]. LCD1 is primary amyloidosis characterized by linear or branching stromal opacities, which stain positive on staining with Congo red and are birefringent under polarized light [3]. GCD2 is characterized by the coexistence of granular deposits and amyloid deposits [4,5]. Corneal opacities of these 3 corneal dystrophies often develop in childhood and gradually progress to cause visual impairment between the third and fifth decades of life. The transforming growth factor β-induced (*TGFBI*) gene, which is expressed in the corneal epithelium and stromal keratocytes, was mapped to the chromosome 5q31 locus [6]. In 1997, Munier et al. [7] reported 4 different missense mutations in *TGFBI* at the CpG dinucleotide of 2 arginine codons: an R555W mutation in a family with GCD1, an R555Q mutation in a family with Reis-Bücklers corneal dystrophy (RBCD), an R124C mutation in 2 families with LCD1, and an R124H mutation in 2 families with GCD2. In retrospect, the phenotype of the family with the R555Q mutation should have been designated as Thiel–Behnke corneal dystrophy (TBCD) instead of RBCD because the original form of RBCD is characterized by confluent geographic opacities, and TBCD is characterized by honeycomb-shaped opacities. RBCD and TBCD are also called corneal dystrophy of Bowman’s layer type 1 (CDB1) and type 2 (CDB2), respectively. These are caused by R124L and R555Q mutations in *TGFBI*, respectively [8,9]. A wide range of variant stromal/Bowman’s layer corneal dystrophies has been found to be associated with different *TGFBI* mutations. At present, more than 50 mutations have been identified in *TGFBI*, with R124 and R555 being the most frequent sites of mutation in various populations. Although a good correlation has been observed between genotype and phenotype—R555W in GCD1, R124C in LCD1, R124H in GCD2, R124L in RBCD, and R555Q in TBCD—some families having corneal dystrophies with *TGFBI* mutations have been reported to have variant phenotypes [10,11]. Clinical presentation may not be sufficient to classify these stromal/Bowman’s layer corneal dystrophies. Hence, screening for mutations in *TGFBI* may facilitate the classification of corneal dystrophies, especially in the cases of atypical clinical presentations.

In this study, we aimed to identify the clinical features and genetic mutation spectrum in patients with *TGFBI*-linked corneal dystrophies from the National Taiwan University Hospital, Taipei, a tertiary-care referral medical center in northern Taiwan. Although genetic studies on *TGFBI*-linked corneal dystrophies have been reported in several populations, no large genetic studies have been reported in the Taiwanese population [12]. The correlations between phenotype and genotype may differ across different ethnic backgrounds; therefore, detection of the characteristics of *TGFBI* mutations in specific populations is important. This may increase our understanding of the clinical–molecular correlations in *TGFBI*.

## Methods

### Subjects

Twenty-five affected patients with corneal dystrophies from 15 unrelated families were recruited from National Taiwan University Hospital. The study was performed in accordance with the tenets of the Declaration of Helsinki and was approved by the Research Ethic Committee of the National Taiwan University Hospital. Informed consent was obtained from the participants before the collection of their peripheral blood. Slit-lamp biomicroscopy and visual acuity and fundus examinations were performed for all participating individuals to determine the disease phenotype. Some of the first-degree relatives were also recruited when available. Two probands with corneal dystrophies received penetrating keratoplasty, and histopathological examinations were performed on their corneal specimens.

### Molecular genetic analysis

Genomic DNA was extracted from the peripheral blood lymphocytes by using the Puregene DNA Purification Blood Kit (Gentra, Minneapolis, MN) according to the manufacturer’s instructions. Each exon of *TGFBI* was amplified by polymerase chain reaction (PCR) by using 50 ng of genomic DNA and a GeneAmp PCR system 9700 thermocycler (Applied Biosystems, Foster City, CA). Primers used for amplifying each exon are listed in Table 1. PCR was performed in 25-μl reaction mixtures containing 20 pmol of each primer, 1× reaction buffer, 100 µM deoxynucleotide triphosphates, and 1 unit of Taq polymerase (Applied Biosystems). Touchdown PCR was performed for all exons except exon 11. Cycling conditions were as follows: initial preheating step at 95 °C for 11 min to achieve a hot start effect, 12 cycles of denaturation at 95 °C for 30 s, initial annealing at 63 °C for 30 s, and extension at 72 °C for 30 s; the annealing temperature was reduced by 0.5 °C per cycle until 56 °C. This was followed by 35 cycles of 95 °C for 30 s, 56 °C for 30 s, and 72 °C for 30 s, and a final extension step at 72 °C for 10 min. The PCR cycling conditions for exon 11 included an initial denaturation at 95 °C for 2 min, followed by 35 cycles of 95 °C for 30 s, 54 °C for 30 s, 72 °C for 30 s, and a final extension step at 72 °C for 7 min. Exons 4 and 12 were sequenced first, followed by the sequencing of exons 11, 13, and 14; the remaining coding exons were sequenced later. The resulting PCR products were purified using the QIAquick PCR Purification Kit (Qiagen, Valencia, CA). Bidirectional sequencing of amplicons was performed using the same PCR forward and reverse primers with the BigDye terminator cycle sequencer kit (Applied Biosystems). The products of the sequencing reaction were analyzed using a fluorescent ABI Prism 3100 DNA sequencer (Perkin Elmer Applied Biosystems, Warrington, UK).

**Table 1:** Polymerase chain reaction primers for the 17 exons of the *TGFBI* gene.

**Exon**	**Sequences of primers**
1	F: 5′-CCGCTCGCAGCTTACTTAAC-3′
	R: 5′-AGCGCTCCATGCTGCAAGGT-3′
2	F: 5′-GTGGACGTGCTGATCATCTT-3′
	R: 5′-TCCTGGCTGGTTACAGATAC-3′
3	F: 5′-GCTGTGGAGGCAACTTAGTG-3′
	R: 5′-GAGAATGCCATGTCCTTGTG-3′
4	F: 5′-CCCCAGAGGCCATCCCTCCT-3′
	R: 5′-CCGGGCAGACGGAGGTCATC-3′
5	F: 5′-TCCTTAGGAAGTGCTGGACT-3′
	R: 5′-CCCCTACCCCATTAGGATAG-3′
6	F: 5′-TGGGCAGATTGTAACTGTGA-3′
	R: 5′-CCCTTACCCGAAGGGTCTCA-3′
7	F: 5′-CCCACAGGCTGCTCTGGCTG-3′
	R: 5′-TGCTCACCTCTCAGGGCTTC-3′
8	F: 5′-ACCCCAGACCTGCTGAACAA-3′
	R: 5′-GGCCTACCTGAGTCTGGGAT-3′
9	F: 5′-CTTGTAGCCAAGAGCACTATT-3′
	R: 5′-ATGTTACCTTTGAATACAGA-3′
10	F: 5′-CTTGTAGATGGAACCCCTCC-3′
	R: 5′-AACTTACATTACGATAAACA-3′
11	F: 5′-TGTGCAGAGCCTCTGCATTG-3′
	R: 5′-TAATTACCTAAAGCGATTGT-3′
12	F: 5′-CATGCTGGTAGCTGCCATCC-3′
	R: 5′-TCTTTACCCAAGAGTCTGCT-3′
13	F: 5′-CCTGCAGGAGATGCCAAGGA-3′
	R: 5′-CACTTACCAAGCTGACTTCC-3′
14	F: 5′-CTTTTAGAAAAACAAATGTG-3′
	R: 5′-CACTTACCTGGAGGCTGCAG-3′
15	F: 5′-TCTTCAGCCAACAGACCTCA-3′
	R: 5′-ATCTTACCCTGGAAAACGCT-3′
16	F: 5′-CTTTCAGGCTTCCCAGAGGT-3′
	R: 5′-GACTCACCTAGTCGCACAGA-3′
17	F: 5′-TTTTCAGCCCCTGTCTATCA-3′
	R: 5′-TATGTTTCTTTGGTTTTATT-3′

### Histopathological examinations

The corneal specimens obtained using penetrating keratoplasty were processed for examination by light microscopy. The tissues were fixed in 10% formalin and embedded in paraffin. The paraffin sections were stained with hematoxylin and eosin (H&E), periodic acid-Schiff (PAS), and Congo red stains.

## Results

### Phenotypes

**Figure 1:**
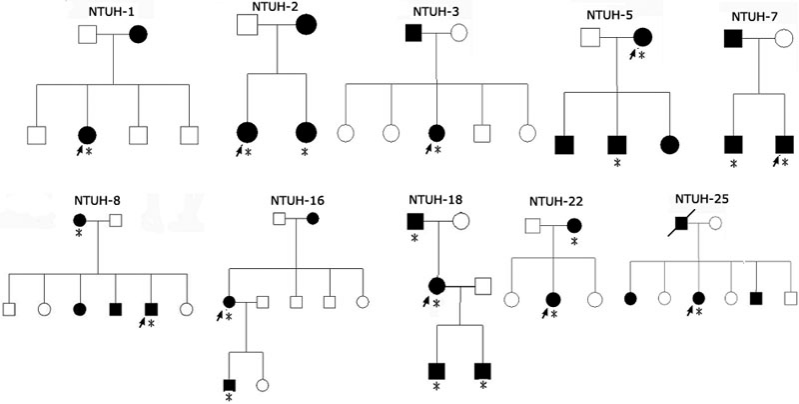
Pedigree of families with *TGFBI*-linked corneal dystrophies. Squares and circles represent male and female participants, respectively. Open symbols indicate unaffected individuals, and solid symbols indicate affected members. Probands are marked by an arrow. Asterisks indicate the members who underwent clinical examination and genetic analysis.

**Figure 2:**
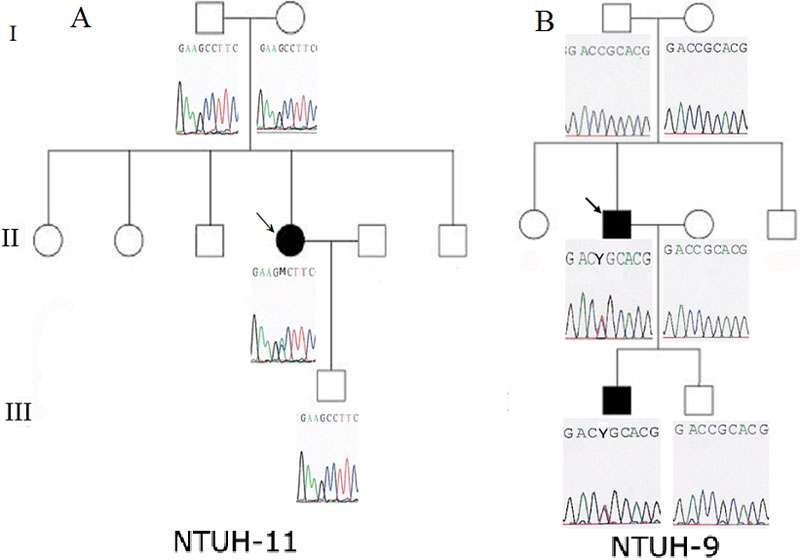
Pedigrees of the NTUH-11 and NTUH-9 families with *TGFBI* sequences. Open symbols indicate unaffected individuals, and solid symbols indicate affected members. Probands are marked by an arrow. **A**: The proband of the NTUH-11 family had a A546D mutation in exon 12, but her parents and son had normal *TGFBI* sequences. **B**: The proband of the NTUH-9 family and his elder son had a heterozygous C→T transition (R124C) in exon 4, but the proband’s parents and his younger son had *TGFBI* sequences without this R124 mutation.

**Figure 3:**
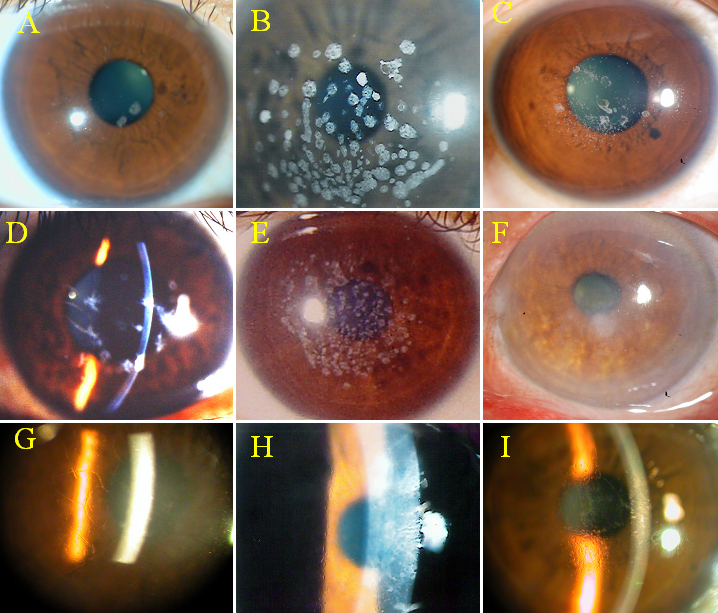
Clinical phenotypes of *TGFBI* corneal dystrophies. **A**: Four round white opacities in the proband of the NTUH-2 family. **B**: Numerous crumb-shaped opacities in the proband of the NTUH-1 family. **C**: Numerous sand-like opacities with some rod-dot granules in the LASIK flap interface in the proband’s younger sister in the NTUH-2 family. **D**: Some dots with thin lines in the proband of the NTUH-6 family. **E**: Superficial breadcrumb-like opacities in the proband of the NTUH-4 family. **F**: Superficial reticular opacities in the proband of the NTUH-18 family. **G**: Flake-dot opacities with lattice-line opacities in the proband of the NTUH-9 family. **H**: Superficially central diffuse haze with some very fine and short lines in the periphery in the proband of the NTUH-15 family. **I**: Numerous small, polymorphic dots with some filamentous lines in the proband of the NTUH-11 family.

Of the 25 affected patients, 22 belonged to 12 families whose pedigrees are shown in Figure 1 and Figure 2, and the remaining 3 (NTUH-4, NTUH-6, and NTUH-15) were sporadic cases. Nine affected subjects from 6 families (NTUH-1, NTUH-2, NTUH-6, NTUH-16, NTUH-22, and NTUH-25) presented with various gray–white granular opacities with or without line opacities. The number of granular opacities ranged from 4 in a 40-year-old female proband of the NTUH-2 family (Figure 3A) to more than 60 in a 26-year-old female proband of the NTUH-1 family (Figure 3B). In the NTUH-2 family, the proband’s younger sister had no remarkable opacities and had normal visual acuity before laser-assisted in situ keratomileusis (LASIK) surgery according to medical records obtained from the referring doctor. She had uncomplicated bilateral LASIK surgery elsewhere. Six months after the operation, some crumb-like opacities and numerous fine confluent opacities developed in the LASIK flap interface, and her visual acuity declined to 20/100 in both eyes (Figure 3C). Most of the crumb-like opacities in these families were elongated or stellate in shape except for some thin lines in the proband, a 57-year-old man, of the NTUH-6 family (Figure 3D). Four affected subjects from 3 families (NTUH-3–5) had superficial bread crumb–like or gray–white granular opacities, which corresponded to GCD1 (Figure 3E). Six affected subjects from 3 families (NTUH-7, NTUH-8, and NTUH-18) presented with reticular-like central superficial corneal opacities in the Bowman’s layer and superficial stroma corresponding to CDB. All the 6 affected subjects had a history of recurrent corneal erosions since early childhood and mild-to-moderate visual impairment. The proband (a 68-year-old man) of the NTUH-18 family received penetrating keratoplasty in the right eye (Figure 3F). The proband (a 40-year-old man) of the NTUH-9 family and his elder son (age, 11 years) showed flake-dot opacities with lattice-line opacities in both eyes (Figure 3G). The parents of the proband of the NTUH-9 family had clear corneas in both the eyes. The proband (a 40-year-old man) of the NTUH-15 family had been experiencing intermittent ocular irritation in both eyes for several years. His corneas showed superficially central diffuse opacities with some very faint fine lines in the periphery, which was thinner and shorter than that observed in the case of typical LCD1 (Figure 3H). He was initially diagnosed as having CDB or atypical LCD1. The proband (a 42-year-old woman) of the NTUH-11 family had small, polymorphic, opaque dots with some filamentous lines in the central cornea (Figure 3I). She had blurry vision since the second decade of life without any episode of corneal erosion and had received penetrating keratoplasty in the left eye. Her parents’ corneas were normal and clear.

### Molecular genetic analysis

**Figure 4:**
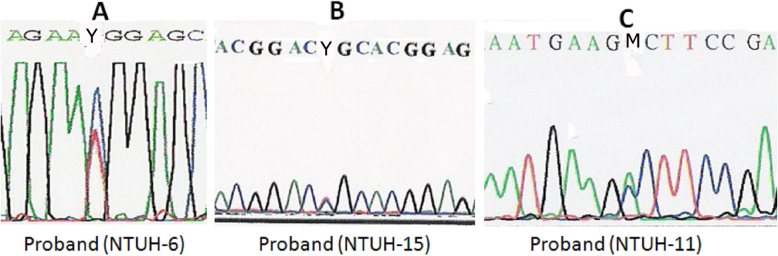
*TGFBI* mutations in 3 families with corneal dystrophies. **A**: A heterozygous C→T transition (R555W) in exon 12 in the proband of the NTUH-6 family. **B**: A heterozygous C→T transition (R124C) in exon 4 in the proband of the NTUH-15 family. **C**: A heterozygous C→A transition (A546D) in exon 12 in the proband of the NTUH-11 family.

Eight affected subjects from 5 families (NTUH-1, NTUH-2, NTUH-16, NTUH-22, and NTUH-25) had an R124H mutation, but the proband of the NTUH-6 family had an R555W mutation (Figure 4A). All 4 affected subjects from the 3 families with GCD1 (NTUH-3–5) had the R555W mutation. Six affected subjects from the 3 families with CDB (NTUH-7, NTUH-8, and NTUH-18) had an R555Q mutation. The proband of the NTUH-15 family with atypical LCD was found to have an R124C mutation rather than the R555W or R124L mutation commonly noted in patients with CDB (Figure 4B). The proband of the NTUH-11 family with polymorphic opacities and some line opacities had an A546D mutation (Figure 4C). Her parents and son did not have this mutation (Figure 2A). The proband of the NTUH-9 family with lattice lines and his affected son had the R124C mutation, but the proband’s parents did not have this mutation in *TGFBI* (Figure 2B). Five distinct *TGFBI* mutations were identified in these 15 families having different subtypes of corneal dystrophies (Table 2).

**Table 2:** Clinical phenotypes and genotypes of the probands of 15 families with *TGFBI*-linked corneal dystrophies.

**Family**	**Phenotype**	**Mutations**	**Age (proband) at examination (years)**	**VA (proband) at examination**
NTUH 1	Numerous granules and some stellate opacities	R124H	26	OD: 0.7; OS: 0.7
NTUH 2	Some granular opacities	R124H	29	OD: 1.0; OS: 0.6 (RD s/p SB)
NTUH 16	Numerous granules and some stellate opacities	R124H	60	OD: 0.05; OS: 0.2 with cataract (OU)
NTUH 22	Numerous granules and some stellate opacities	R124H	30	OD: 1.0; OS: 1.0
NTUH 25	Numerous granules and some stellate opacities	R124H	68	OD: 0.5; OS: 0.5 with cataract (OU)
NTUH 6	Some granules with slim lines	R555W	52	OD: 0.5; OS: 0.6
NTUH 3	Bread crumb opacities	R555W	39	OD: 0.5; OS: 0.6
NTUH 4	Bread crumb opacities	R555W	24	OD: 0.4; OS: 0.3
NTUH 5	Bread crumb opacities	R555W	45	OD: 0.5; OS: 0.5
NTUH 7	Reticular superficial opacities	R555Q	21	OD: 0.6; OS: 0.6
NTUH 8	Reticular superficial opacities	R555Q	57	OD: 0.1; OS: 0.1 with cataract (OU)
NTUH 18	Reticular superficial opacities	R555Q	68	OD: 0.05; OS: 0.05 with cataract (OU)
NTUH 9	Flake-dot opacities with lattice lines	R124C	36	OD: 0.3; OS: 0.4
NTUH 15	Superficially diffuse haze with some fine lines	R124C	32	OD: 0.4; OS: 0.4
NTUH 11	Polymorphic dots with lattice lines	A546D	39	OD: 0.4; OS: 0.2

OD: right eye, OS: left eye, OU: both eyes. RD s/p SB: retinal detachment after surgery with scleral buckle.

### Histopathological examinations

**Figure 5:**
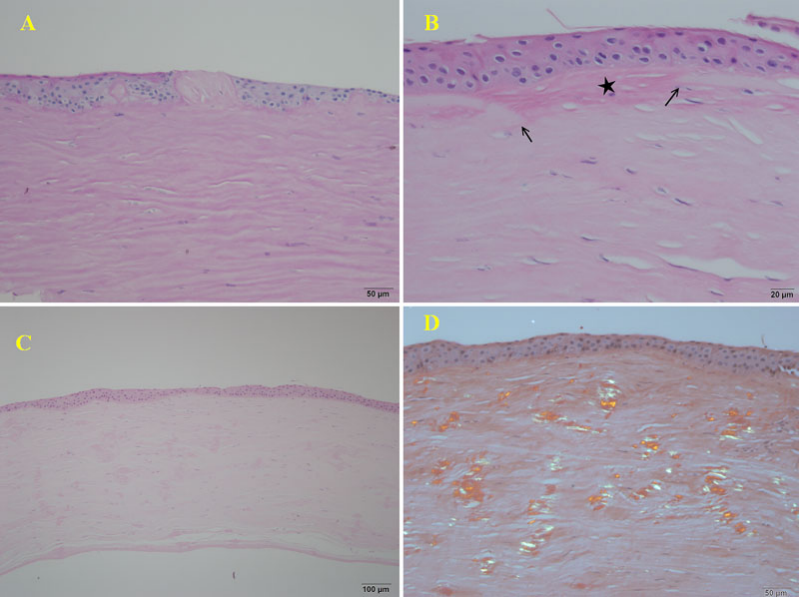
Histopathology. **A**: The corneal specimen from the proband of the NTUH-18 family showed irregular thickness of the epithelium, vacuolization in the basal epithelium, and focal subepithelial fibrosis interposed between the irregular epithelium with a “sawtooth-like” configuration (PAS staining, 200×). **B**: Focal disruption of Bowman’s membrane (arrows) was replaced by subepithelial fibrotic tissue (star) (H&E staining, 400×). **C**: The specimen from the proband of the NTUH-11 family showed several eosinophilic deposits interspersed within the entire corneal stromal layer (H&E staining, 200×). **D**: These deposits showed green birefringence under a polarized microscope (Congo red staining, 200×).

Microscopic examination of the corneal specimens obtained using penetrating keratoplasty in the proband of the NTUH-18 family showed irregular epithelial thickness, vacuolization in the basal epithelium, focal disruption of the Bowman’s layer, and undulating subepithelial fibrosis (Figure 5A,B). Corneal specimens obtained after penetrating keratoplasty in the proband of the NTUH-11 family showed numerous eosinophilic deposits interspersed within the entire stromal layer (Figure 5C). These deposits showed apple-green birefringence on Congo red staining under polarized light, corresponding to amyloid deposits (Figure 5D).

## Discussion

Mutations in *TGFBI* have often been identified in several different stromal/Bowman’s layer corneal dystrophies, including in GCD1, GCD2, RBCD (CDB1), TBCD (CDB2), LCD1, and atypical LCDs [13]. The classic GCD1 with the R555W mutation presents as multiple small white spots in the Bowman’s layer and superficial corneal stroma. Most of our patients with the R555W mutation showed the typical features of GCD1 noted worldwide; however, the proband of the NTUH-6 family showed dot and line opacities, which have rarely been reported in GCD1 with the R555W mutation. The vision of patients with the R555W mutation was mildly affected, with a range between 20/30 and 20/50. The discrete round opacities in our GCD2 patients with the R124H mutation were larger than those noted in classical GCD1 and occasionally coexisted with line opacities, sharing some features with GCD1 and LCD. GCD2 is usually linked with the R124H mutation and has considerable intra- and interfamilial phenotypic variation. Wide interfamilial variation was also observed in our 5 GCD2 families. The proband of the NTUH-2 family had few granular deposits without any line opacities, unlike the other 4 families with GCD2. In fact, this proband could have been misdiagnosed as having GCD1 because GCD2, like GCD1, might show an initial presentation of a few granular deposits. Our results indicated that there was a good phenotype–genotype correlation in most patients with GCD1 and GCD2, but phenotypic variation was noted in some cases.

Exacerbation of corneal opacities after LASIK was observed in the younger sister of the proband of the NTUH-2 family with GCD2. Several reports have shown rapid acceleration of granular corneal opacities after LASIK in patients with GCD2 [14,15]. The increased deposits often appeared in the flap interface and mainly within the ablation zone, suggesting that an increased production of TGFBI protein by keratocytes in the region of corneal trauma was related to lamellar corneal incision and excimer laser ablation. Hence, we recommend that patients with GCD2 or any other *TGFBI*-linked corneal dystrophies should not be considered for LASIK surgery because the corneal opacities in the interface might increase, and the vision may worsen after the operation. Detailed examination of corneas and ensuring no overlooking of any bilaterally subtle corneal opacity in the patients referred for LASIK surgery is important. Further genetic analysis of *TGFBI* in cases in which corneal dystrophy is suspected can help identify patients with atypical GCD2 and prevent this adverse event from occurring after LASIK surgery.

Patients with corneal opacities at the level of Bowman’s layer have previously been diagnosed as having CDB, but some of them were thought to have a superficial variant of granular corneal dystrophy [16]. Typical RBCD (CDB1) and TBCD (CDB2) are characterized by geographic opacities and honeycomb-shaped opacities, respectively. Most patients reported to have CDB1 had the R124L mutation and those reported to have CDB2 had the R555Q mutation [17,18]. However, the R124L and R555Q mutations do not account for all forms of CDB. Other mutations may cause similar phenotypes, including a ΔF540 mutation described in a Sardinian family and a G623D mutation [19,20]. In addition, Yee et al. [21] reported a family with TBCD showing “peculiar curly” filaments in the sub-epithelial layer of the cornea. This trait was mapped to chromosome 10q23–24 instead of the *TGFBI* locus at 5q31. Although these reports suggest the phenotypic and genetic heterogeneity inherent in CDB, our study in the 6 patients of our 3 CDB families indicated that patients with TBCD would have characteristic presentation of honeycomb corneal opacities at the Bowman’s layer and superficial stroma, and were correlated with the R555Q mutation. Histology of these honeycomb corneal opacities was an undulating fibrous tissue in the subepithelium and focal disruption of the Bowman’s membrane. However, histology may not be available in all patients with CDB. Genetic study of *TGFBI* can be accurate to classify these CDB because of a good genotype-phenotype correlation in TBCD/R555Q.

LCD1 is characterized by a network of delicate interdigitating filaments within the corneal stroma. The disease usually begins in the first decade of life with symptoms of recurrent painful epithelial erosions. Lattice lines and diffuse opacification of the central cornea develop gradually after the erosions and amyloid accumulations. The most common mutation in *TGFBI* in patients with LCD1 is R124C. Numerous forms of atypical LCD have been reported to be caused by P501T, V505D, L518P, I522N, L527R, V539D, A546D, A546T, P551Q, L569R, H572R, V625D, or H626R mutations [22-27]. The proband of the NTUH-11 family had the A546D mutation and blurred vision, with no history of recurrent corneal erosion. She presented with polymorphic refractile dots and filamentous lines in the deep stroma, which were unlike those found in typical LCD1. The histological examination showed amyloidal deposits in the entire stromal layer. These findings were similar to the cases of polymorphic corneal amyloidosis first described by Eifrig et al. [25]. However, 2 reports revealed that the A546D mutation could also cause phenotypes of either atypical LCD or GCD1 [28,29]. Reports of the A546D mutation in *TGFBI* have been rare. This mutation might result in different clinical phenotypes.

Interestingly, the proband of the NTUH-15 family with the R124C mutation had diffuse central grayish opacities in the subepithelium and superficial stroma without typical branching refractile lattice lines that are characteristic of LCD1. Unfortunately no histology was available in this proband, but these superficial opacities were more similar to CDB than to typical LCD1. Several studies have reported similar findings. For instance, a Chinese family with RBCD had the R124C mutation instead of the common R124L mutation, and a family from New Zealand with atypical CDB had the H626P mutation, a known mutation linked to variant LCD [30,31]. These results indicate that phenotypic variabilty may occur in patients with R124C or H626P mutations, which segregate with phenotypes of either CDB or LCD. The superficial stromal opacities in this proband with the R124C mutation were not like classic TBCD, RBCD, or LCD1. This atypical presentation may be due to interaction between *TGFBI* and other genes or the effect of environmental factors on gene presentation.

Spontaneous mutations in the *TGFBI* gene have previously been reported, including an R124L mutation in 2 patients with RBCD and an R555Q mutation in 2 families with CDB [32,33]. In our study, spontaneous mutations were found in 2 families, one with an R124C mutation and another with an A546D mutation. In addition, the spontaneously mutated allele could be transmitted to the next generation. Most of the reported spontaneous mutations in the *TGFBI* gene are at the 2 common hotspots, namely, R124 and R555, and involve a G:C→A:T transition more frequently than an A:T→G:C transition [34]. Spontaneous mutation of A546D with an A→C transition has not yet been reported.

Our study showed that codons R124 and R555 of *TGFBI* were the 2 mutational hotspots in autosomal dominant corneal dystrophies in Taiwan, as they were in other ethnic groups. Interestingly, the predominant mutations varied across different countries. For example, the classic GCD1/R555W mutation is the most prevalent mutation in Europe. The GCD2/R124H mutation is the most common mutation in Japan and Korea [35,36]. The GCD1/R555W and GCD2/R124H mutations are the 2 most common mutations in China. The GCD1/R555W and LCD1/R124C mutations are the 2 most common mutations in India [37]. The LCD/H626R and GCD1/R555W mutations are the 2 most common mutations in Mexico [38]. The GCD1/R555W is the most common mutation in New Zealand [39]. The pattern of *TGFBI* mutations showed some correlations among these Northeast Asian countries and India, but not Vietnam, LCD/H626R more predominant than LCD1/R124C [40]. In our study, the GCD2/R124H and GCD1/R555W mutations were the 2 most common mutations in Taiwan, as has been reported in China. This may be due to the shared ancestry between the Taiwanese and Chinese populations. Interestingly, the TBCD/R555Q mutation (3/15) in our study was not rare as in China (1/64) or Japan (6/286). This may be related to the founder effect, a bias of a small sample size, the influence of admixture between the Taiwanese ancestral population and the local South Polynesian population, or spontaneous *TGFBI* mutations. Phylogenetic trees and correspondence analysis calculated from human leukocyte antigens allele frequencies have shown that Taiwanese have a more affinity to southern Asian population than northern Han Chinese or Japanese [41]. Our results of *TGFBI* mutations in Taiwanese were correspondence to these findings. A close relationship between Taiwan indigenous people and Oceanians, and 13% of Taiwan indigenous genes in Taiwanese gene pool were also found [42]. It may explain this unique result of *TGFBI* mutations in Taiwanese. Further large-scale studies involving more Taiwanese families with *TGFBI*-linked corneal dystrophies are required to confirm our preliminary findings and the speculation of the relationship between Taiwanese and other Asian population. More reports on *TGFBI* mutations from other Southeast Asian or Pacific Ocean countries might help clarify the difference between ethnic background and genotypes and understand their possible relationship across different countries.

In conclusion, a good phenotype–genotype correlation was observed in most patients with *TGFBI*-linked corneal dystrophies. Intra- and interfamilial phenotypic variation occurred occasionally. Genetic screening of *TGFBI* might facilitate precise clinical diagnosis and corneal dystrophy classification, especially in patients with atypical presentation.
